# Sex-Specific Cognitive Divergence in Parkinson’s Disease

**DOI:** 10.3390/brainsci16070764

**Published:** 2026-07-21

**Authors:** Julia Saltyte Benth, Gonzalo Sanchez Nido, Kenn Freddy Pedersen, Ole Bjørn Tysnes, Guido Werner Alves, Charalampos Tzoulis, Geir Olve Skeie

**Affiliations:** 1 Neuro-SysMed, Department of Neurology, Haukeland University Hospital, 5021 Bergen, Norway; gonzalo.nido@uib.no (G.S.N.); ole-bjorn.tysnes@helse-bergen.no (O.B.T.); charalampos.tzoulis@uib.no (C.T.); geir.skeie@uib.no (G.O.S.); 2 Department of Clinical Medicine, University of Bergen, P.O. Box 7804, 5020 Bergen, Norway; 3 K.G. Jebsen Center for Translational Research in Parkinson’s Disease, University of Bergen, P.O. Box 7804, 5020 Bergen, Norway; 4 Department of Neurology, Lillehammer Hospital, Innlandet Hospital Trust, 2690 Lillehammer, Norway; kenn.freddy.pedersen@sykehuset-innlandet.no; 5 Department of Neurology and Center for Movement Disorders, Stavanger University Hospital, 4068 Stavanger, Norway; 6 Department of Chemistry, Bioscience and Environmental Engineering, University of Stavanger, 4036 Stavanger, Norway

**Keywords:** Parkinson’s disease, cognition, MCI, dementia, sex differences, long-term changes

## Abstract

**Highlights:**

**What are the main findings?**

- In this 9-year longitudinal study, Parkinson’s disease-related sex-specific divergence was restricted to semantic fluency and processing speed.
- Males with Parkinson’s disease exhibit faster domain-specific cognitive decline rather than a generalized vulnerability.

**What are the implications of the main findings?**

- Our findings have implications for cognitive monitoring in Parkinson’s disease.
- The findings support further investigation of sex-specific mechanisms underlying cognitive heterogeneity in Parkinson’s disease.

**Abstract:**

*Background:* Sex differences in cognitive impairment in Parkinson’s disease (PD) have been reported, with several studies suggesting greater cognitive decline in males. However, evidence on long-term trajectories across specific cognitive domains is limited, as most prior work is cross-sectional. *Aim:* To investigate sex-specific longitudinal changes across multiple cognitive domains over 9 years in idiopathic PD, compared to age- and sex-matched controls. *Methods:* We analyzed data from the prospective ParkWest study, including n = 190 persons with PD (PwPs) and n = 202 controls. Regularly assessed cognitive domains included semantic fluency, processing speed, cognitive control, memory, and visuospatial ability. Linear mixed-effect models including a three-way interaction between time, disease status, and sex, to test whether sex-specific cognitive change in PD differed from that observed in controls, were estimated. Models were adjusted for age, years of education, baseline depression, and UPDRS part II. *Results:* Male PwPs showed faster decline than female PwPs in most cognitive measures, resulting in gradually increasing sex differences over 9 years. However, compared with controls, the differential sex-specific decline was statistically significant only for semantic fluency (*p* = 0.013) and processing speed (*p* = 0.027 and *p* = 0.035). These findings remained significant after covariate adjustment. *Conclusions:* PD was associated with increasing sex differences in semantic fluency and processing speed, likely driven by faster decline in males. Other domains did not show sex-specific divergence over time, suggesting that sex-specific vulnerabilities in cognitive trajectories are domain-specific rather than generalized among PwPs.

## 1. Introduction

Male sex is associated with a greater overall risk of developing Parkinson’s disease (PD) and with a different disease phenotype trajectory than females [1]. Males generally have more rigidity, bradykinesia, freezing, falls, and cognitive impairment, while females exhibit more tremor and treatment-induced dyskinesias and less cognitive dysfunction. Moreover, previous studies have indicated different cognitive profiles in male and female persons with PD (PwPs), suggesting that PD affects cognition differently across sexes.

Cross-sectional studies have shown inconsistent results, with reports of poorer cognition in either sex [2,3,4] or no differences [5]. Interpretation of these studies is compounded by the use of cognitive screening tools with limited resolution (e.g., MMSE [6]), cross-sectional designs which do not capture longitudinal progression, and frequent absence of appropriately matched controls.

Few studies attempted to capture PD-related cognitive phenotypes, using domain-specific batteries to assess executive function, processing speed, memory, and visuospatial ability. Although results vary, a tentative pattern has emerged in which females with PD tend to perform worse on visuospatial measures but better on memory tasks [7,8], whereas males more often show deficits in processing speed [9,10,11] and aspects of executive function, including fluency [5,12]. However, the strength and consistency of these associations vary greatly across cohorts, and most studies rely on cross-sectional designs.

Longitudinal studies focusing on cognitive sex differences in PD are sparse and inconclusive. Some studies suggest that males may be at higher risk for more rapid progression to PD with mild cognitive impairment (MCI) and dementia, but they often lack control groups and do not characterize trajectories within specific cognitive domains [9,13]. Another longitudinal study that included controls and specific cognitive domains found modest sex-related differences in cognitive trajectories that did not diverge over time; however, the follow-up period was short [7].

Overall, the research field supports the existence of sex-specific cognitive profiles in PD, but their nature, extent, PD-specificity, and longitudinal evolution remain largely undetermined. Addressing this need requires long-term, controlled studies with repeated, domain-sensitive cognitive assessments. The aim of this study was thus to investigate how idiopathic PD affects cognitive trajectories in males and females compared with normal controls for 9 years of follow-up. We suggest that sex-specific vulnerabilities in cognitive trajectories in PD are domain-specific.

## 2. Materials and Methods

### 2.1. Study Design

The longitudinal ParkWest cohort study [14] was used to assess sex differences in cognitive symptoms among PwPs. Data from the first 9 years of follow-up (inclusion between 2004 and 2006, last follow-up between 2013 and 2015) were analyzed in the present study. Excluding PwPs who were re-diagnosed to conditions other than PD or atypical PD, the final population consisted of n = 190 PwPs (115 males and 75 females) and n = 202 age- and sex matched normal controls (106 males and 96 females).

To minimize environmental and socioeconomic confounders, spouses of PwPs were recruited as controls, when possible. All motor and non-motor assessments of participants with PD were conducted at their “On” state of medication. Disease duration before diagnosis was determined by the difference between initial PD symptoms and time of diagnosis. Participants regarded as demented at baseline were excluded. We used the Montgomery and Aasberg Depression Rating Scale (MADRS) to assess depressive symptoms among participants at baseline. Biological sex was assigned based on X/Y chromosome genotyping.

Disease severity was assessed by the sum of part I, II, and III of the original Unified Parkinson’s Disease Rating Scale (UPDRS). Since motor-subtypes have been shown to affect sex and cognition differently, we classified PwPs to Postural Instability and Gait Disorder PD (PIGD-PD) or Tremor Dominant PD (TD-PD), both according to UPDRS part III [11,14,15]. This classification is plausible given that PIGD-PD has been shown to be associated with faster cognitive decline than TD-PD [11].

As cognitive decline has been found to predict later conversion to MCI and PDD, we classified ParkWest participants with probable MCI and PDD according to the MDS criteria [16,17] for our exploratory analyses. The MCI diagnosis was estimated in both PwPs and controls and based on the following criteria: (i) a z-score ≤ 1.5 on 2 or more of the 8 specific cognitive tests (Table 1). Z-scores were calculated as the difference between the observed value for a given participant and average of controls, divided by standard deviation of controls, thereby generating study-specific rather than published normative values. Correlations among cognitive tests were not accounted for. (ii) A score of >3.0 on the Informant Questionnaire on Cognitive Decline in the Elderly or a score of ≥1 on the intellectual impairment item of the UPDRS part I. A diagnosis of PDD was assigned by neurologists (for more details, see Pedersen et al. [17]). Cases of PDD and MCI at baseline and 1, 3, 5, 7, and 9 years of follow-up were reported.

### 2.2. Confounders

Potential confounding variables were preselected according to literature [3,5,11,16,18] and clinical knowledge. These included age, years of education, baseline depression, and UPDRS part II. Age and years of education are known to affect cognitive performance, and so is depression. However, depression was not recorded longitudinally in the ParkWest study, and thus only baseline status was included. Since daily functioning and cognition may evolve together over time, the UPDRS part II was included as a time-varying confounder assessed simultaneously with the outcomes. Other parts of the UPDRS and other disease-specific covariates could not be included as confounders, as these were assessed only for PwPs in our material.

### 2.3. Evolution of Cognitive Ability

Cognitive ability was evaluated by employing 9 different tests at baseline and 1, 3, 5, 7, and 9 years of follow-up. (i) MMSE [6] was used to assess global cognition. In addition, 8 tests were performed to assess specific cognitive aspects: (ii) Verbal Category Fluency (VCF) [19], (iii–v) the three-fold Stroop Color and Word Test (SCWT) [11,20], (vi–vii) the immediate and delayed recall of the California Verbal Learning Test II (CVLT-II) [17], and (viii–ix) subtest 2 and 8 of Visuospatial Object and Space Perception (Vosp) [5]. In all tests, higher scores correspond to better cognition. These specific tests are described in Table 1 and were classified according to 3 cognitive domains [16].

### 2.4. Statistical Analysis

Between- and within-group comparisons of participants were performed by Pearson’s χ^2^-test, Fisher’s exact test, and Welch Two-Sample *t*-test. Numerical variables were summarized by means and standard deviations (SDs), while categorical variables were summarized by frequencies and percentages.

To assess sex-specific differences in the decline of cognitive ability during 9 years among PwPs and to account for intraindividual correlations over time, we estimated linear mixed-effects models (LMMs), one for each cognitive test. First, we estimated an unadjusted model

1a. *y ~ time × disease status × sex*,

and then a model adjusting for potential confounders, including age, years of education (*yoe*), depression at baseline (*dab*), and UPDRS part II (*updrs*)

1b. *y ~ time × disease status × sex + age + yoe + dab + updrs*.

The three-way interaction was considered our primary outcome. Both models (1a and 1b) included also all possible two-way interactions between time (in years), disease status (PwPs vs. controls), and sex (male vs. female), together with random intercepts for participants. Random slope for time was also considered. Although this slightly improved model fit according to Akaike’s Information Criterion, the results were altered only marginally. We opted therefore to favor more parsimonious models and did not include a random slope term.

Potential non-linear associations between outcome variables and numerical covariates (age, years of education, UPDRS part II, and time) were assessed by including squared covariates and applying Akaike’s Information Criterion and Bayesian Information Criterion. Only UPDRS part II exhibited a non-linear relationship with MMSE, VCF, Vosp (both subparts), and the word-naming part of SCWT, and therefore, an additional quadratic term for UPDRS part II was included in LMMs. LMM partially accounts for missing values due to participants that were lost to follow-up throughout the study (n = 65 PwPs and n = 63 normal controls (Supplementary Table S1)) by including all available information up to last follow-up. To assess potential attrition bias, age, sex, depression status, years of education, and baseline UPDRS part II status and cognitive performance were compared between dropouts and non-dropouts at 5- and 9-year follow-up by Pearson’s χ^2^-test and Welch Two-Sample *t*-test.

Multicollinearity between confounders was examined by pairwise correlations. Model fit was assessed with marginal and conditional R^2^ and visual inspection of histogram of residuals. Effect sizes were graphically illustrated as standardized regression coefficients with corresponding 95% confidence intervals (CIs).

As an exploratory analysis, sex- and disease-specific differences in MCI and sex-specific differences in PDD were presented by Kaplan–Meier curves, and groups were compared with a log-rank test.

We used two-sided *p* < 0.05 to denote nominal statistical significance. Because the preliminary analyses involved multiple cognitive outcomes, we additionally applied the Benjamini–Hochberg procedure to adjust for multiple testing across the 9 primary three-way interaction tests. This analysis is intended to assess the robustness of nominally significant findings to multiplicity adjustment. A false discovery rate (FDR) threshold of 0.20 was used, which we considered appropriate given our aim to capture a broader understanding of cognitive trajectories. In addition, for all tests performed, we present the results as effect sizes with the corresponding 95% CIs or standard errors (SEs), preserving the transparency. Both nominal *p*-values and FDR-adjusted significance thresholds (i.e., Benjamini–Hochman critical values) for primary outcomes are presented. All analyses were performed in R studio (R version 4.4.1).

## 3. Data Sharing

The data that support the findings of this study are available on request from the corresponding author. The data are not publicly available due to privacy or ethical restrictions.

## 4. Results

Baseline characteristics of the included 190 PwPs and 202 normal controls are presented in Table 2 and Tables S2–S4. As expected, there were more males among the PwPs (n = 115 (60.5%)), which was balanced by recruitment of age- and sex-matched controls.

PwPs had significantly fewer years of education compared with normal controls (*p* = 0.001), and so did females as compared with males in the PD group (*p* = 0.002). Overall, PwPs (n = 27 (14.2%)) had more depressive symptoms than controls (n = 9 (4.5%), (*p* < 0.001)). Among PwPs, males showed a longer disease duration before diagnosis compared to females (*p* = 0.03), but there were no significant differences in disease severity (total UPDRS), neither at baseline nor after 9 years of follow-up (Supplementary Table S2, Supplementary Figure S1). This also applied to each UPDRS-part (I-IV) (Supplementary Figures S1 and S2). At baseline, distribution of PIGD-PD and TD-PD did not differ between males and females.

Eligible cases at six time points are presented in Supplementary Table S1. According to Pearson’s χ^2^-test, there were no differences between PwPs and controls in dropout rate. Comparison of dropouts and non-dropouts showed no differences in sex nor depression status. Dropouts were significantly older among both PwPs (*p* < 0.001 at 5- and 9-year follow-up) and controls (*p* = 0.049 and *p* < 0.001 at 5- and 9-year follow-up, respectively). In addition, dropouts among controls were slightly less educated (*p* = 0.024 and *p* = 0.001 at 5- and 9-year follow-up, respectively) and UPDRS part II status was significantly worse among PwPs dropouts (*p* = 0.001 and *p* < 0.001 at 5- and 9-year follow-up, respectively). With respect to cognitive status at baseline, significant differences between dropouts and non-dropouts in performance were found for nearly all cognitive tests, with controls performing numerically better regardless of dropout status.

**Figure 1:**
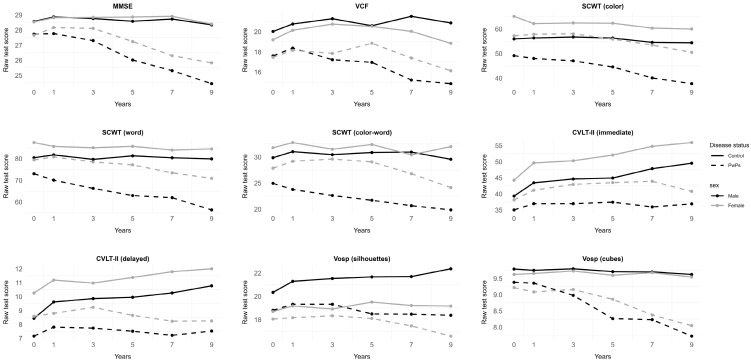
Cognitive test battery. Graphic illustration of cognitive trajectories (MMSE, VCF, CVLT-II, Vosp, and SCWT) over 9 years in males and females, with and without Parkinson’s disease.

Figure 1 and Supplementary Table S5 present descriptive numbers for each test. The results of LMMs for all cognitive scores are summarized in Supplementary Tables S6–S14 and illustrated in Figure 1, Figures S3 and S4. Cognitive ability declined significantly faster in PwPs compared with controls across all specific cognitive domains, and this difference persisted after adjusting for potential confounders (i.e., significant time-by-disease status interaction). A similar decline in performance was observed on the MMSE, although statistical significance was lost after adjustment.

**Figure 2:**
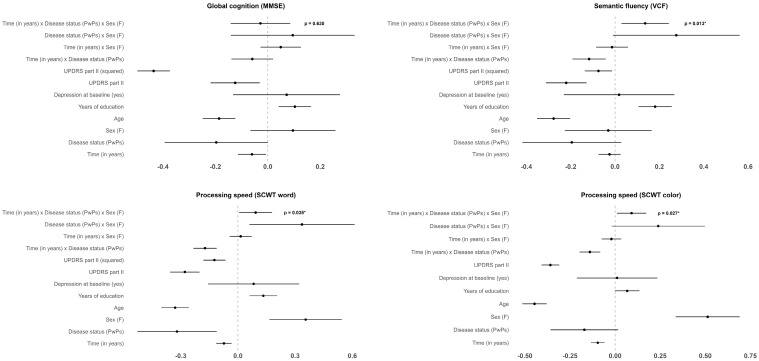
Scaled coefficient plots. Scaled output of linear mixed-effect models (MMSE, VCF, SCWT color and word). * Significant at significance level 0.05.

The primary analysis focusing on the time-by-disease status-by-sex interaction indicated that sex differences widened more over time in PwPs than in controls for VCF (*p* = 0.013, β = 0.27, SE = 0.11) and congruent SCWT (color: *p* = 0.027, β = 0.45, SE = 0.20, and word: *p* = 0.035, β = 0.56, SE = 0.27); see Figure 2 for effect sizes with the corresponding 95% CIs. All these three-way interactions remained significant after FDR-adjustment (significance thresholds of 0.022, 0.044, and 0.066, respectively). These differences were mainly driven by faster decline in males and were only found for these measures. In contrast, for the incongruent SCWT, the three-way interaction was not significant, while there was evidence of an overall sex-specific difference (*p* = 0.019, β = 3.67, SE = 1.56 for the group-by-sex interaction) in the adjusted model (Supplementary Table S14). None of the other cognitive domains showed evidence of faster decline in males as no significant three-way interactions were identified for these measures. Sex-specific longitudinal effects were thus restricted to a limited subset of cognitive measures, suggesting domain-specific vulnerability. These findings were consistent in unadjusted and adjusted models. Moreover, there were moderate to high correlations within the three-folded SCWT and between SCWT and VCF (Supplementary Figure S5).

As part of the exploratory analysis, Supplementary Tables S15 and S16 summarize the distribution of MCI and PDD. The proportion of PwPs with MCI increased from 30.5% (29.6% males, 32.0% females) at baseline to 47.3% (53.7% males, 37.8% females) at 9-year follow-up. Among normal controls, corresponding numbers were 7.4% (11.9% males, 2.3% females) at baseline and 9.6% (10.4% males and 8.3% females) at 9-year follow-up. According to the Kaplan–Meier curves (Supplementary Figure S6), the risk for MCI was lower among females independent of disease status, with *p* < 0.001 for global log-rank test. Although, pairwise comparisons revealed that this difference was due to disease- rather than sex-specific differences.

While no cases of PDD were reported at 1-year follow-up, the frequencies steadily increased from 7.5% (5.6% males, 10.6% females) at 3 years to 32.0% (38.7% males, 18.3% females) at 9 years. Kaplan–Meier curves (Supplementary Figure S7) indicated slightly lower risk of PDD in females as compared to males. This sex-specific difference was, however, only significant at a 10% level (*p* = 0.065 for log-rank test).

## 5. Discussion

In this 9-year longitudinal study of the ParkWest cohort, we investigated whether PD is associated with sex-specific cognitive trajectories beyond those observed in normal aging. PwPs showed consistently poorer performance and faster decline than controls across all specific cognitive domains. However, PD-related sex-specific divergence was restricted to semantic fluency and processing speed, where male PwPs declined faster than female PwPs relative to the corresponding sex differences observed in controls. This could not be attributed to age, years of education, depression at baseline, or daily functioning as measured by the UPDRS part II. These findings suggest that sex-specific cognitive vulnerability in PD is domain-specific rather than generalized.

Our results are consistent with previous studies suggesting poorer performance among male PwPs in semantic fluency and processing speed [5,9,10,11,12]. Most earlier studies, however, have been cross-sectional or had shorter follow-up times, limiting their ability to distinguish between baseline sex differences and divergence over time. The present study extends this knowledge by showing that sex differences in semantic fluency and processing speed become more pronounced during long-term follow-up in PD, and that this pattern is stronger than the sex-related differences observed in controls.

Worsening cognitive performance in semantic fluency and processing speed may partly reflect the increasing burden of cognitive impairment in the cohort over time. However, exploratory analyses did not show clear sex-specific differences in risk of MCI or PDD, likely because of less advanced PD. Yet, these findings should be interpreted cautiously, as unlike Pedersen et al. [17], MCI classification was based on study-specific z-scores rather than published normative data. This approach allows a consistent method across all cognitive tests and maximizes sensitivity for detecting cognitive change, but it also limits comparability with studies using external norms. Conversely, published normative data are not available for all cognitive measures included in our battery and are often derived from populations that differ from ours in demographic and clinical characteristics, making their consistent application challenging.

Bayram et al. [7] reported cognitive trajectories in PD that were similar to healthy aging when stratified by sex and MCI status, which aligns with our findings except for semantic fluency and processing speed. Differences in follow-up duration (4 vs. 9 years), cohort composition (stratification by MCI status), cognitive test batteries, covariate handling, and statistical modeling may account for this discrepancy. Our results are also consistent with Cholerton et al. [9], who found that males advanced faster to PD-MCI and PDD over 8 years of follow-up, although they did not assess long-term trajectories in specific cognitive domains. Taken together, these findings suggests that males and females exhibit different cognitive profiles as PD progresses but only limited to some cognitive domains.

The mechanisms underlying sex-specific cognitive trajectories in PD remain uncertain. Executive function [18] and informative processing speed [21] have been linked to cognitive reserve (CR), which is commonly proxied by educational attainment alongside factors such as occupational complexity and premorbid intelligence [11,18]. Because only educational attainment was available, we could only assess CR partially by adjusting for years of education in our analyses. Consequently, we cannot fully account for the potential influence of CR. Nevertheless, evidence for sex differences in CR is scant and mixed, and available longitudinal data suggest limited impact of CR on progression to PD-MCI [21,22]. Notably, sex differences in semantic fluency and processing speed have been reported in studies that matched males and females on factors contributing to CR, suggesting that CR alone cannot explain the observed pattern [11].

On the other hand, prior studies have proposed that early cognitive decline in PD may be at least partly attributed to nigrostriatal dopamine loss and impairment of the frontostriatal circuits [9,18,23], affecting attention [24], executive function [24], and processing speed [23]. Estrogen-related neuroprotection of dopaminergic circuits has been suggested as one possible contributor to sex differences in these domains [9,25]. Although not assessed in our cohort, given the age at onset of our PwPs, estrogen-related protection would likely be negligible already at baseline in most female participants [1,25]. Depression may also affect cognition and has been proposed as a neurofunctional factor influencing cognitive performance [3,5,16], potentially through shared pathways with attention, executive function, and processing speed [7,26,27]. We adjusted for depressive symptoms at baseline but did not address later occurrences of depression. While depression did not have a significant effect on either cognitive measure in our study, in line with a previous report [5], later depressive symptoms may still have contributed to observed changes in semantic fluency and processing speed [26]. These potential mechanisms remain speculative and should be examined in future longitudinal studies.

Motor phenotype may also contribute to cognitive heterogeneity in PD and show a different degree of cognitive influence and sex-dependent variation in expression. The PIGD phenotype is, compared to TD, associated with a more profound cognitive impairment [28] and has been shown to be dominated by males [11]. However, we found no sex differences in baseline phenotype distributions in our idiopathic PD cohort. This indicates that phenotype could not account for the faster decline in semantic fluency and processing speed tasks in male PwPs in our study, in line with Reekes et al. [11].

Our study has some limitations. First, semantic fluency can be category-dependent [29]; ParkWest assessed only the “animals” category, precluding evaluation across categories. Second, impairment of semantic fluency in PD may partly reflect bradyphrenia (slowed thinking) rather than executive dysfunction [30]. We did not formally test whether reduced processing speed accounted for the semantic fluency findings, as this was beyond the scope of this work. Third, attrition (largely due to death) may have introduced survival bias and informative dropout, potentially violating the assumption of missingness at random, which the LMM relies on. Sex, as out main covariate, was similarly distributed among dropouts and non-dropouts in both controls and PwPs. With respect to cognitive outcomes, dropouts among both PwPs and controls were cognitively worse for nearly all tests. Moreover, controls outperformed PwPs overall. Further, compared to non-dropouts, all dropouts were older, controls were slightly less educated and PwPs had worse UPDRS part II status. Because these covariates are known to be associated with poorer cognitive function and were included as confounders, some bias may have been mitigated. Additionally, the selective loss of participants with poorer cognitive status suggests that identified differences are likely conservative, i.e., in case of no dropouts they could potentially become more pronounced. Nevertheless, as with all longitudinal studies affected by attrition, these findings should be interpreted with caution and replicated by other studies. Fourth, although our three-way interactions were considered our main outcome, the possibility of false positive results may occur. Therefore, we transparently present all the performed analyses. Moreover, the results within cognitive domains clearly point the same direction and are in line with existing cross-sectional studies [11]. Furthermore, the Benjamini–Hochberg adjustment confirmed our main findings, supporting the robustness of the results. Fifth, even though including the spouse of a respective PD participant is expected to minimize environmental differences, the reduction in environmental variability could also affect generalizability. Finally, other confounding factors besides those accounted for in this study may be associated with the dependent variables, especially sleep-related factors such as excessive daytime sleepiness and REM behavior disorder, which could affect processing speed and executive functions [31]. However, these were not preselected confounders. In addition, they likely correlate with depression and UPDRS part II, both included in the LMMs, causing potential multicollinearity issues and complicating already complex models. Nevertheless, their implications for our study should only remain speculative as they were not formally tested, and future studies should further investigate their contribution to sex differences in cognitive trajectories in PD.

## 6. Conclusions

While our findings do not support a generalized faster cognitive decline in male PwPs, they suggest a domain-specific vulnerability in males limited to semantic fluency and processing speed. As a result, sex differences among PwPs in these domains increased over time beyond what is expected by healthy aging alone and remained robust after Benjamini–Hochberg adjustment for multiple testing. Our findings may have implications for cognitive monitoring in PD and support further investigation of sex-specific mechanisms underlying cognitive heterogeneity in the disease.

## Figures and Tables

**Table 1:** Cognitive tests used in this study.

	Test	Cognitive Assessment	Description
Global cognition	MMSE ^1^	General cognition	Orientation, verbal questions, calculations, language, drawing, and writing
Specific cognitive domains:			
I. Executive functions	VCF ^2^	Semantic fluency	Verbal animal-naming within 1 min
	Congruent SCWT ^3^ (color and word)	Processing speed	Recite color and color-words from a given list within 45 s
	Incongruent SCWT ^3^ (color-word)	Cognitive control	Recite the color of color-words in mismatching colors from a given list within 45 s
II. Memory	CVLT-II ^4^ (List A)	Memory	Recall verbally listed words immediately afterwards and after a delay of 20 min
III. Visuospatial ability	Vosp ^5^ (silhouettes and cubes)	Perception of spatial relations	Distinguish animal-silhouettes, identify number of cubes

^1^ MMSE: Mini-Mental State Examination. ^2^ VCF: Verbal Category Fluency. ^3^ SCWT: Stroop Color and Word Test. ^4^ CVLT-II: Californian Verbal Learning Test part II. ^5^ Vosp: Visuospatial Object and Space Perception.

**Table 2:** Baseline characteristics.

	Persons with Parkinson’s Disease			Controls			Overall		
Characteristic	Male n = 115 ^1^	Female n = 75 ^1^	*p*-Value ^2^	Male n = 106 ^1^	Female n = 96 ^1^	*p*-Value ^3^	Persons with Parkinson’s Disease n = 190 ^1^	Control n = 202 ^1^	*p*-Value ^2^
Age (years)	67.3 (9.3)	69.1 (9.1)	0.2	66.1 (10.2)	66.5 (8.8)	0.7	68.0 (9.3)	66.3 (9.6)	0.070
Education (years)	11.7 (3.4)	10.2 (2.9)	0.002	12.7 (3.5)	11.8 (3.9)	0.12	11.1 (3.3)	12.3 (3.7)	0.001
Depression at baseline	19 (17%)	8 (11%)	0.3	3 (3%)	6 (6%)	0.3	27 (14%)	9 (5%)	<0.001
Disease duration before diagnosis	2.5 (1.9)	1.9 (1.4)	0.032						
PD Phenotype			0.2						
PIGD ^4^	44 (38%)	36 (48%)							
Indeterminate	17 (15%)	5 (7%)							
TD ^5^	54 (47%)	34 (45%)							

^1^ Mean (SD); n (%); ^2^ Welch Two-Sample *t*-test; Pearson’s χ^2^; ^3^ Welch Two-Sample *t*-test; Fisher’s exact test; ^4^ Postural Instability and Gait Disorder (PIGD); ^5^ Tremor dominant (TD).

## Data Availability

The data that support the findings of this study are available on request from the corresponding author. The data are not publicly available due to privacy or ethical restrictions.

## Supplementary Materials

The following supporting information can be downloaded at: https://www.mdpi.com/article/10.3390/brainsci16070764/s1, Figure S1: Unified Parkinsons Disease Rating Scale. Graphic illustration of UPDRS part II and total score-trajectory over 9 years in males and females, with and without PD; Figure S2: Unified Parkinsons Disease Rating Scale. Graphic illustration of UPDRS part I, III and IV score-trajectory over 9 years in male and female PwPs; Figure S3: Scaled coefficient plots. Scaled output of linear mixed-effect models (CVLT-II and Vosp); Figure S4: Scaled coefficient plot. Scaled output of linear mixed-effect model (SCWT colour-word); Figure S5: Pairwise correlations. Pairwise correlations of the cognitive test battery; Figure S6: Kaplan-Meier curve. The share of males and females with and without Parkinson’s disease without mild cognitive impairment (MCI); Figure S7: Kaplan-Meier curve. The share of male and female PwPs with no Parkinson’s disease associated dementia; Table S1: Eligible cases at each follow-up year; Table S2: Score on the UPDRS (part I, III, IV and total); Table S3: Score on the UPDRS (part II); Table S4: Score on the UPDRS (part II); Table S5: Performance on cognitive test battery; Table S6: Analysis of cognitive performance (MMSE); results of linear mixed-effect model; Table S7: Analysis of cognitive performance (VCF); results of linear mixed-effect model; Table S8: Analysis of cognitive performance (CVLT-II, List A–immediate recall); results of linear mixed-effect model; Table S9: Analysis of cognitive performance (CVLT-II, List A–delayed recall); results of linear mixed-effect model; Table S10: Analysis of cognitive performance (Vosp-silhouettes); results of linear mixed-effect model; Table S11: Analysis of cognitive performance (Vosp-cube); results of linear mixed-effect model; Table S12: Analysis of cognitive performance (SCWT-color); results of linear mixed-effect model; Table S13: Analysis of cognitive performance (SCWT-word); results of linear mixed-effect model; Table S14: Analysis of cognitive performance (SCWT–color-word); results of linear mixed-effect model; Table S15: Distribution of Parkinson’s disease-associated dementia (PDD); Table S16: Distribution of mild cognitive impairment (MCI).
