# Supplementary material for: Sex-Specific Cognitive Divergence in Parkinson’s Disease
Source: Brain Sci. 2026 Jul 21;16(7):764. doi: 10.3390/brainsci16070764 (PMC13407224; doi:10.3390/brainsci16070764)
Supplement: Supplementary file 1 [file brainsci-16-00764-s001.zip › brainsci-4418272-supplementary.pdf]

Table S1 – Eligible cases at each follow-up year:

	Persons with Parkinson's Disease		Controls		Overall	
**Time point (year)**	**Male**	**Female**	**Male**	**Female**	**Persons with Parkinson's disease**	**Controls**
**Baseline**	115	75	106	96	190	202
**1**	113	71	101	90	184	191
**3**	108	66	95	85	174	180
**5**	97	61	88	79	158	167
**7**	86	57	80	72	143	152
**9**	75	50	72	67	125	139

Table S2 – Score on the UPDRS (part I, III, IV and total):

	Baseline			9 Years		
**Test**	**Male** n = 115[1]	**Female** n = 75[1]	*p-value*[2]	**Male** n = 75[1]	**Female** n = 50[1]	*p-value*[2]
**UPDRS part I**	1.9 (1.8)	2.1 (1.8)	0.751	4.1 (3.7)	3.9 (3.5)	0.869
**UPDRS part III**	24.3 (10.7)	22.5 (12.4)	0.292	31.5 (18.2)	27.8 (15.7)	0.224
**UPDRS part IV**	0.5 (1.2)	0.6 (1.0)	0.343	3.3 (2.6)	3.6 (2.6)	0.447
**total UPDRS[3]**	35.0 (14.7)	33.8 (17.9)	0.616	52.8 (29.8)	47.7 (24.6)	0.306

[1] Mean (SD)

[2] Welch Two-Sample t-test

[3] As a sum of part I, II and III

Table S3 – Score on the UPDRS (part II):

	Baseline						9 Years					
	Persons with Parkinson's disease			Controls			Persons with Parkinson's disease			Controls		
**Test**	**Male**[1] n = 115	**Female**[1] n = 75	*p-value*[2]	**Male**[1] n = 106	**Female**[1] n = 96	*p-value*[2]	**Male**[1] n = 75	**Female**[1] n = 50	*p-value*[2]	**Male**[1] n = 72	**Female**[1] n = 67	*p-value*[2]
**UPDRS part II**	8.7 (4.5)	9.2 (5.8)	0.522	0.5 (1.0)	0.6 (1.4)	0.805	17.1 (9.5)	15.9 (7.5)	0.431	1.3 (2.5)	2.4 (4.6)	0.099

[1] Mean (SD)

[2] Welch Two-Sample t-test

Table S4 – Score on the UPDRS (part II):

	Baseline			9 Years		
**Test**	**Persons with Parkinson's disease**[1] n = 190	**Controls**[1] n = 202	*p-value*[2]	**Persons with Parkinson's disease**[1] n = 125	**Controls**[1] n = 139	*p-value*[2]
**UPDRS part II**	8.9 (5.1)	0.5 (1.2)	< 0.001	16.7 (8.7)	1.8 (3.7)	< 0.001

Table S5 – Performance on cognitive test battery:

	Baseline				9 Years			
	Persons with Parkinson's disease		Controls		Persons with Parkinson's disease		Controls	
**Cognitive test**	**Male**[1] n = 115	**Female**[1] n = 75	**Male**[1] n = 106	**Female**[1] n = 96	**Male**[1] n = 75	**Female**[1] n = 50	**Male**[1] n = 72	**Female**[1] n = 67
**Global cognition (MMSE[2])**	27.7 (2.3)	27.7 (2.6)	28.6 (1.5)	28.5 (1.4)	24.5 (6.8)	25.8 (6.2)	28.3 (2.4)	28.4 (2.4)
**Semantic fluency (VCF[3])**	17.6 (5.9)	17.5 (5.1)	20.0 (5.8)	19.2 (5.2)	14.9 (7.1)	16.1 (5.9)	20.9 (6.2)	18.9 (5.8)
**Immediate recall (CVLT-II[4], List A)**	35.1 (11.6)	38.2 (11.9)	39.4 (11.3)	44.3 (11.7)	36.9 (16.6)	40.8 (15.9)	49.5 (13.9)	56.0 (14.4)
**Delayed recall (CVLT-II[4], List A)**	7.2 (3.8)	8.6 (3.7)	8.4 (3.5)	10.3 (3.2)	7.5 (4.9)	8.3 (4.4)	10.8 (3.7)	12.0 (3.9)
**Visuospatial abilities (Vosp[5] Silhouettes)**	18.8 (3.9)	18.1 (4.6)	20.3 (3.6)	18.7 (3.8)	18.4 (5.1)	16.6 (5.4)	22.4 (4.1)	19.2 (4.5)
**Visuospatial abilities (Vosp[5] Cubes)**	9.4 (1.2)	9.2 (1.3)	9.8 (0.5)	9.6 (1.1)	7.7 (3.3)	8.0 (2.7)	9.6 (1.1)	9.5 (1.4)
**Processing speed (SCWT[6] Colour-naming)**	49.2 (13.6)	57.2 (15.6)	56.0 (11.8)	65.1 (12.2)	37.8 (17.9)	50.5 (17.3)	54.4 (12.9)	60.0 (13.2)
**Processing speed (SCWT[6] Word-naming)**	73.1 (18.2)	79.4 (18.4)	80.5 (14.8)	87.5 (14.5)	56.4 (25.8)	71.0 (19.2)	79.9 (15.8)	84.5 (16.2)
**Cognitive control (SCWT[6] Colour-words)**	24.9 (11.2)	27.8 (12.3)	29.9 (10.3)	31.8 (9.8)	19.9 (13.1)	24.1 (12.7)	29.6 (10.6)	32.0 (9.7)

[1] Mean (SD)

	Baseline				9 Years			
	Persons with Parkinson's disease		Controls		Persons with Parkinson's disease		Controls	
**Cognitive test**	**Male**[1] n = 115	**Female**[1] n = 75	**Male**[1] n = 106	**Female**[1] n = 96	**Male**[1] n = 75	**Female**[1] n = 50	**Male**[1] n = 72	**Female**[1] n = 67

[2] MMSE: Mini-Mental State Examination

[3] VCF: Verbal Category Fluency

[4] CVLT-II: Californian Verbal Learning Test part II

[5] Vosp: Visuospatial Object and Space Perception

[6] SCWT: Stroop Colour and Word Test

Table S6 – Analysis of cognitive performance (MMSE); results of linear mixed-effect model

	**Interaction only**				**Adjusted model**			
Predictors	*Estimates*	*SE*[1]	*CI*[2]	*p*	*Estimates*	*SE*[1]	*CI*[2]	*p*
Time	-0.05	0.03	-0.12 – 0.01	0.095	-0.06	0.03	-0.12 – -0.01	**0.027**
Disease status (PwPs[3])	-0.63	0.36	-1.33 – 0.07	0.079	-0.39	0.34	-1.06 – 0.27	0.248
Sex (F[4])	-0.01	0.37	-0.74 – 0.73	0.985	0.10	0.31	-0.50 – 0.70	0.736
Time * Disease status (PwPs[3])	-0.41	0.05	-0.50 – -0.32	**<0.001**	-0.06	0.04	-0.14 – 0.02	0.145
Time * Sex (F[4])	0.03	0.05	-0.06 – 0.13	0.468	0.05	0.04	-0.03 – 0.13	0.209
Disease status (PwPs[3]) * Sex (F[4])	0.03	0.54	-1.03 – 1.10	0.949	0.44	0.45	-0.44 – 1.31	0.328
Time * Disease status (PwPs[3]) * Sex (F[4])	0.12	0.07	-0.02 – 0.25	0.091	-0.03	0.06	-0.15 – 0.09	0.630
Age					-0.07	0.01	-0.09 – -0.04	**<0.001**
Years of education					0.10	0.03	0.04 – 0.15	**0.001**
Depression at baseline (Yes)					0.24	0.34	-0.43 – 0.91	0.488
UPDRS part II					0.10	0.03	0.04 – 0.16	**0.002**
UPDRS part II (squared)					-0.01	0.00	-0.01 – -0.01	**<0.001**
Intra-correlation coefficient	0.46				0.42			
N	392 $_{case}$				392 $_{case}$			
Observations	1966				1966			
Marginal R^2 / Conditional R^2	0.153 / 0.546				0.392 / 0.645			

[1] Standard Error (SE)
[2] Confidence Interval (CI)
[3] Persons with Parkinson's disease (PwPs)
[4] Female (F)

Table S7 – Analysis of cognitive performance (VCF); results of linear mixed-effect model

	Interaction only				Adjusted model			
Predictors	*Estimates*	*SE*[1]	*CI*[2]	*p*	*Estimates*	*SE*[1]	*CI*[2]	*p*
Time	-0.06	0.05	-0.16 – 0.04	0.249	-0.05	0.05	-0.15 – 0.05	0.324
Disease status (PwPs[3])	-2.07	0.74	-3.52 – -0.61	**0.005**	-0.26	0.72	-1.67 – 1.15	0.715
Sex (F[4])	-0.44	0.78	-1.97 – 1.08	0.567	-0.08	0.67	-1.39 – 1.23	0.903
Time * Disease status (PwPs[3])	-0.52	0.07	-0.66 – -0.37	**<0.001**	-0.23	0.08	-0.38 – -0.08	**0.002**
Time * Sex (F[4])	-0.06	0.07	-0.21 – 0.08	0.414	-0.03	0.07	-0.17 – 0.11	0.712
Disease status (PwPs[3]) * Sex (F[4])	0.14	1.13	-2.07 – 2.36	0.899	0.62	0.97	-1.29 – 2.53	0.527
Time * Disease status (PwPs[3]) * Sex (F[4])	0.39	0.11	0.17 – 0.60	**<0.001**	0.27	0.11	0.06 – 0.48	**0.013**
Age					-0.18	0.03	-0.23 – -0.13	**<0.001**
Years of education					0.32	0.07	0.19 – 0.45	**<0.001**
Depression at baseline (Yes)					0.11	0.79	-1.44 – 1.67	0.887
UPDRS part II					-0.14	0.06	-0.25 – -0.03	**0.016**
UPDRS part II (squared)					-0.00	0.00	-0.01 – -0.00	**0.017**
Intra-correlation coefficient	0.66				0.58			
N	391_{case}				391_{case}			
Observations	1952				1952			
Marginal R^2 / Conditional R^2	0.095 / 0.690				0.267 / 0.691			

[1] Standard Error (SE)

[2] Confidence Interval (CI)
[3] Persons with Parkinson's disease (PwPs)
[4] Female (F)

Table S8 – Analysis of cognitive performance (CVLT-II, List A – immediate recall); results of linear mixed-effect model

	**Interaction only**				**Adjusted model**			
Predictors	*Estimates*	*SE*[1]	*CI*[2]	*p*	*Estimates*	*SE*[1]	*CI*[2]	*p*
Time	0.63	0.10	0.43 – 0.82	**<0.001**	0.68	0.10	0.49 – 0.87	**<0.001**
Disease status (PwPs[3])	-4.37	1.77	-7.83 – -0.90	**0.014**	1.78	1.56	-1.27 – 4.83	0.254
Sex (F[4])	5.19	1.85	1.57 – 8.82	**0.005**	6.08	1.56	3.02 – 9.14	**<0.001**
Time * Disease status (PwPs[3])	-1.05	0.14	-1.33 – -0.77	**<0.001**	-0.40	0.15	-0.68 – -0.11	**0.007**
Time * Sex (F[4])	0.32	0.14	0.03 – 0.60	**0.028**	0.42	0.14	0.15 – 0.69	**0.003**
Disease status (PwPs[3]) * Sex (F[4])	-1.64	2.69	-6.92 – 3.63	0.541	-0.84	2.27	-5.29 – 3.62	0.712
Time * Disease status (PwPs[3]) * Sex (F[4])	-0.08	0.22	-0.51 – 0.34	0.699	-0.35	0.21	-0.76 – 0.06	0.094
Age					-0.54	0.06	-0.65 – -0.42	**<0.001**
Years of education					0.61	0.16	0.29 – 0.93	**<0.001**
Depression at baseline (Yes)					-2.03	1.90	-5.75 – 1.70	0.286
UPDRS part II					-0.64	0.05	-0.74 – -0.54	**<0.001**
Intra-correlation coefficient	0.76				0.69			
N	390 $_{case}$				390 $_{case}$			
Observations	1915				1915			
Marginal R^2 / Conditional R^2	0.148 / 0.792				0.360 / 0.804			

[1] Standard Error (SE)
[2] Confidence Interval (CI)
[3] Person's with Parkinson's disease (PwPs)
[4] Female (F)

Table S9 – Analysis of cognitive performance (CVLT-II, List A – delayed recall); results of linear mixed-effect model

	**Interaction only**				**Adjusted model**			
Predictors	*Estimates*	*SE*[1]	*CI*[2]	*p*	*Estimates*	*SE*[1]	*CI*[2]	*p*
Time	0.12	0.03	0.06 – 0.18	**<0.001**	0.13	0.03	0.07 – 0.19	**<0.001**
Disease status (PwPs[3])	-1.25	0.50	-2.24 – -0.26	**0.014**	0.31	0.45	-0.57 – 1.20	0.487
Sex (F[4])	1.62	0.53	0.59 – 2.66	**0.002**	1.85	0.45	0.97 – 2.73	**<0.001**
Time * Disease status (PwPs[3])	-0.28	0.04	-0.36 – -0.19	**<0.001**	-0.12	0.04	-0.20 – -0.03	**0.009**
Time * Sex (F[4])	-0.01	0.04	-0.10 – 0.08	0.810	0.02	0.04	-0.07 – 0.10	0.702
Disease status (PwPs[3]) * Sex (F[4])	-0.50	0.77	-2.01 – 1.01	0.519	-0.27	0.66	-1.55 – 1.02	0.686
Time * Disease status (PwPs[3]) * Sex (F[4])	-0.02	0.07	-0.14 – 0.11	0.814	-0.08	0.06	-0.21 – 0.04	0.201
Age					-0.16	0.02	-0.19 – -0.13	**<0.001**
Years of education					0.15	0.05	0.06 – 0.24	**0.001**
Depression at baseline (Yes)					-0.38	0.54	-1.45 – 0.69	0.483
UPDRS part II					-0.16	0.02	-0.19 – -0.13	**<0.001**
Intra-correlation coefficient	0.73				0.66			
N	390 case				390 case			
Observations	1908				1908			
Marginal R^2 / Conditional R^2	0.125 / 0.764				0.323 / 0.770			

1 Standard Error (SE)
2 Confidence Interval (CI)
3 Person's with Parkinson's disease (PwPs)

[4] Female (F)

Table S10 – Analysis of cognitive performance (Vosp - silhouettes); results of linear mixed-effect model

	**Interaction only**				**Adjusted model**			
Predictors	*Estimates*	*SE[1]*	*CI[2]*	*p*	*Estimates*	*SE[1]*	*CI[2]*	*p*
Time	0.13	0.03	0.07 – 0.20	**<0.001**	0.13	0.03	0.07 – 0.19	**<0.001**
Disease status (PwPs[3])	-1.52	0.55	-2.61 – -0.43	**0.006**	-0.64	0.52	-1.66 – 0.38	0.218
Sex (F[4])	-1.82	0.58	-2.96 – -0.68	**0.002**	-1.59	0.50	-2.56 – -0.62	**0.001**
Time * Disease status (PwPs[3])	-0.38	0.04	-0.47 – -0.29	**<0.001**	-0.17	0.05	-0.26 – -0.08	**<0.001**
Time * Sex (F[4])	-0.14	0.05	-0.23 – -0.05	**0.002**	-0.12	0.04	-0.21 – -0.04	**0.005**
Disease status (PwPs[3]) * Sex (F[4])	0.98	0.85	-0.68 – 2.64	0.248	1.36	0.72	-0.05 – 2.78	0.059
Time * Disease status (PwPs[3]) * Sex (F[4])	0.10	0.07	-0.03 – 0.24	0.140	0.01	0.07	-0.12 – 0.14	0.892
Age					-0.17	0.02	-0.21 – -0.14	**<0.001**
Years of education					0.17	0.05	0.07 – 0.27	**0.001**
Depression at baseline (Yes)					-0.00	0.60	-1.19 – 1.18	0.995
UPDRS part II					-0.02	0.04	-0.09 – 0.05	0.608
UPDRS part II (squared)					-0.01	0.00	-0.01 – -0.00	**<0.001**
Intra-correlation coefficient	0.75				0.69			
N	391 $_{case}$				391 $_{case}$			
Observations	1952				1952			
Marginal R^2 / Conditional R^2	0.110 / 0.776				0.311 / 0.785			

[1] Standard Error (SE)
[2] Confidence Interval (CI)
[3] Person's with Parkinson's disease (PwPs)
[4] Female (F)

Table S11 – Analysis of cognitive performance (Vosp - cube); results of linear mixed-effect model

	Interaction only				Adjusted model			
Predictors	*Estimates*	*SE*[1]	*CI*[2]	*p*	*Estimates*	*SE*[1]	*CI*[2]	*p*
Time	-0.02	0.02	-0.05 – 0.02	0.298	-0.02	0.02	-0.05 – 0.02	0.355
Disease status (PwPs[3])	-0.30	0.18	-0.65 – 0.06	0.099	0.17	0.19	-0.21 – 0.55	0.384
Sex (F[4])	-0.14	0.19	-0.51 – 0.23	0.464	-0.13	0.17	-0.46 – 0.21	0.460
Time * Disease status (PwPs[3])	-0.21	0.03	-0.26 – -0.16	**<0.001**	-0.07	0.03	-0.12 – -0.02	**0.005**
Time * Sex (F[4])	0.01	0.03	-0.04 – 0.06	0.714	0.02	0.02	-0.03 – 0.07	0.404
Disease status (PwPs[3]) * Sex (F[4])	-0.05	0.27	-0.58 – 0.49	0.868	-0.01	0.25	-0.49 – 0.48	0.977
Time * Disease status (PwPs[3]) * Sex (F[4])	0.06	0.04	-0.02 – 0.13	0.139	-0.01	0.04	-0.08 – 0.07	0.871
Age					-0.02	0.01	-0.03 – -0.01	**<0.001**
Years of education					-0.00	0.02	-0.04 – 0.03	0.769
Depression at baseline (Yes)					-0.24	0.19	-0.60 – 0.13	0.200
UPDRS part II					-0.02	0.02	-0.06 – 0.01	0.230
UPDRS part II (squared)					-0.00	0.00	-0.00 – -0.00	**<0.001**
Intra-correlation coefficient	0.38				0.34			
N	391 $_{case}$				391 $_{case}$			
Observations	1946				1946			
Marginal R^2 / Conditional R^2	0.144 / 0.473				0.265 / 0.516			

[1] Standard Error (SE)
[2] Confidence Interval (CI)
[3] Person's with Parkinson's disease (PwPs)
[4] Female (F)

Table S12 – Analysis of cognitive performance (SCWT - color); results of linear mixed-effect model

	Interaction only				Adjusted model			
Predictors	*Estimates*	*SE*[1]	*CI*[2]	*p*	*Estimates*	*SE*[1]	*CI*[2]	*p*
Time	-0.51	0.10	-0.71 – -0.32	**<0.001**	-0.47	0.09	-0.65 – -0.29	**<0.001**
Disease status (PwPs[3])	-6.61	1.86	-10.26 – -2.97	**<0.001**	0.19	1.47	-2.69 – 3.08	0.895
Sex (F[4])	7.83	1.94	4.02 – 11.65	**<0.001**	8.38	1.47	5.50 – 11.26	**<0.001**
Time * Disease status (PwPs[3])	-1.44	0.14	-1.71 – -1.16	**<0.001**	-0.68	0.14	-0.96 – -0.40	**<0.001**
Time * Sex (F[4])	-0.22	0.14	-0.50 – 0.06	0.118	-0.10	0.13	-0.36 – 0.16	0.462
Disease status (PwPs[3]) * Sex (F[4])	0.54	2.83	-5.01 – 6.09	0.849	1.86	2.14	-2.34 – 6.05	0.385
Time * Disease status (PwPs[3]) * Sex (F[4])	0.72	0.21	0.31 – 1.14	**0.001**	0.45	0.20	0.05 – 0.84	**0.027**
Age					-0.73	0.06	-0.85 – -0.62	**<0.001**
Years of education					0.29	0.15	-0.00 – 0.59	0.052
Depression at baseline (Yes)					0.17	1.76	-3.28 – 3.63	0.922
UPDRS part II					-0.76	0.05	-0.87 – -0.66	**<0.001**
Intra-correlation coefficient	0.79				0.68			
N	388 $_{case}$				388 $_{case}$			
Observations	1905				1905			
Marginal R^2 / Conditional R^2	0.232 / 0.836				0.487 / 0.836			

[1] Standard Error (SE)
[2] Confidence Interval (CI)
[3] Person's with Parkinson's disease (PwPs)
[4] Female (F)

Table S13 – Analysis of cognitive performance (SCWT - word); results of linear mixed-effect model

	**Interaction only**				**Adjusted model**			
Predictors	*Estimates*	*SE*[1]	*CI*[2]	*p*	*Estimates*	*SE*[1]	*CI*[2]	*p*
Time	-0.49	0.13	-0.74 – -0.23	**<0.001**	-0.44	0.12	-0.69 – -0.20	**<0.001**
Disease status (PwPs[3])	-8.12	2.28	-12.59 – -3.64	**<0.001**	-1.81	2.05	-5.83 – 2.22	0.379
Sex (F[4])	5.71	2.40	1.02 – 10.41	**0.017**	6.47	1.95	2.65 – 10.29	**0.001**
Time * Disease status (PwPs[3])	-2.14	0.19	-2.51 – -1.78	**<0.001**	-1.04	0.19	-1.41 – -0.67	**<0.001**
Time * Sex (F[4])	-0.04	0.19	-0.41 – 0.33	0.827	0.09	0.18	-0.26 – 0.44	0.625
Disease status (PwPs[3]) * Sex (F[4])	2.13	3.48	-4.69 – 8.95	0.541	4.13	2.83	-1.42 – 9.68	0.145
Time * Disease status (PwPs[3]) * Sex (F[4])	0.96	0.28	0.41 – 1.52	**0.001**	0.56	0.27	0.04 – 1.08	**0.035**
Age					-0.67	0.07	-0.82 – -0.53	**<0.001**
Years of education					0.72	0.20	0.33 – 1.12	**<0.001**
Depression at baseline (Yes)					1.59	2.34	-3.01 – 6.18	0.498
UPDRS part II					-0.48	0.15	-0.79 – -0.18	**0.002**
UPDRS part II (squared)					-0.02	0.01	-0.03 – -0.01	**<0.001**
Intra-correlation coefficient	0.75				0.68			
N	391 $_{case}$				391 $_{case}$			
Observations	1929				1929			
Marginal R^2 / Conditional R^2	0.216 / 0.805				0.415 / 0.812			

[1] Standard Error (SE)
[2] Confidence Interval (CI)
[3] Person's with Parkinson's disease (PwPs)
[4] Female (F)

Table S14 – Analysis of cognitive performance (SCWT – color-word); results of linear mixed-effect model

	**Interaction only**				**Adjusted model**			
Predictors	*Estimates*	*SE*[1]	*CI*[2]	*p*	*Estimates*	*SE*[1]	*CI*[2]	*p*
Time	-0.28	0.08	-0.43 – -0.12	**<0.001**	-0.25	0.07	-0.40 – -0.11	**0.001**
Disease status (PwPs[3])	-5.68	1.43	-8.49 – -2.87	**<0.001**	-1.10	1.08	-3.22 – 1.02	0.310
Sex (F[4])	1.81	1.50	-1.13 – 4.75	0.228	2.31	1.07	0.20 – 4.41	**0.032**
Time * Disease status (PwPs[3])	-0.79	0.11	-1.01 – -0.58	**<0.001**	-0.30	0.11	-0.52 – -0.08	**0.008**
Time * Sex (F[4])	0.05	0.11	-0.16 – 0.27	0.633	0.14	0.11	-0.07 – 0.35	0.190
Disease status (PwPs[3]) * Sex (F[4])	2.26	2.18	-2.01 – 6.54	0.299	3.67	1.56	0.60 – 6.73	**0.019**
Time * Disease status (PwPs[3]) * Sex (F[4])	0.11	0.17	-0.22 – 0.44	0.516	-0.07	0.16	-0.39 – 0.24	0.644
Age					-0.63	0.04	-0.71 – -0.55	**<0.001**
Years of education					0.34	0.11	0.13 – 0.56	**0.002**
Depression at baseline (Yes)					0.69	1.27	-1.81 – 3.18	0.590
UPDRS part II					-0.50	0.04	-0.59 – -0.42	**<0.001**
Intra-correlation coefficient	0.78				0.63			
N	385 $_{case}$				385 $_{case}$			
Observations	1887				1887			
Marginal R^2 / Conditional R^2	0.150 / 0.813				0.482 / 0.809			

[1] Standard Error (SE)
[2] Confidence Interval (CI)
[3] Person's with Parkinson's disease (PwPs)

[4] Female (F)

Table S15 – Distribution of Parkinson's disease-associated dementia (PDD)

Time point (year)	Male	Female	Overall
**1**	0 (-)<br>n = 113	0 (-)<br>n = 71	0 (-)<br>n = 184
**3**	6 (5.6%)<br>n = 108	7 (10.6%)<br>n = 66	13 (7.5%)<br>n = 174
**5**[2]	18 (18.6%)<br>n = 97	9 (14.8%)<br>n = 61	27 (17.1%)<br>n = 158
**7**[3]	24 (27.9%)<br>n = 86	8 (14.0%)[1]<br>n = 57	32 (22.4%)<br>n = 143
**9**[4]	29 (38.7%)<br>n = 75	11 (18.3%)<br>n = 50	40 (32.0%)<br>n = 125

[1] n = 2 females with PDD were among dropouts at 7 years, and 1 new case of PDD appeared, explaining the reduction in cases of PDD
[2] n = 2 controls reported as demented
[3] n = 6 controls reported as demented
[4] n = 6 controls reported as demented

Table S16 – Distribution of mild cognitive impairment (MCI)

	Persons with Parkinson's disease		Controls		Overall	
**Time point (year)**	**Male**[1]	**Female**[1]	**Male**[1]	**Female**[1]	**PwPs**[1]	**C**[1]
**0**	34 (29.6%)<br>n = 115	24 (32.0%)<br>n = 75	12 (11.9%)<br>n = 101	2 (2.3%)<br>n = 87	58 (30.5%)<br>n = 190	14 (7.4%)<br>n = 188
**1**	40 (35.4%)<br>n = 113	15 (21.4%)<br>n = 70	10 (10.4%)<br>n = 96	7 (8.9%)<br>n = 79	55 (30.1%)<br>n = 183	17 (9.7%)<br>n = 175
**3**	47 (44.3%)<br>n = 106	15 (24.2%)<br>n = 62	9 (10.1%)<br>n = 89	5 (6.8%)<br>n = 74	62 (36.9%)<br>n = 168	14 (8.6%)<br>n = 163
**5**	37 (40.2%)<br>n = 92	12 (20.7%)<br>n = 58	7 (8.9%)<br>n = 78	9 (14.3%)<br>n = 63	49 (32.7%)<br>n = 150	16 (11.3%)<br>n = 141
**7**	37 (46.8%)<br>n = 79	21 (38.9%)<br>n = 54	6 (8.5%)<br>n = 71	4 (8.5%)<br>n = 47	58 (43.6%)<br>n = 133	10 (8.5%)<br>n = 118
**9**	36 (53.7%)<br>n = 67	17 (37.8%)<br>n = 45	7 (10.4%)<br>n = 67	4 (8.3%)<br>n = 48	53 (47.3%)<br>n = 112	11 (9.6%)<br>n = 115

[1] n (%): number of cases assessed

Reducing percentages in time because of dropout and lack of criteria-fulfilment for proper MCI assessment

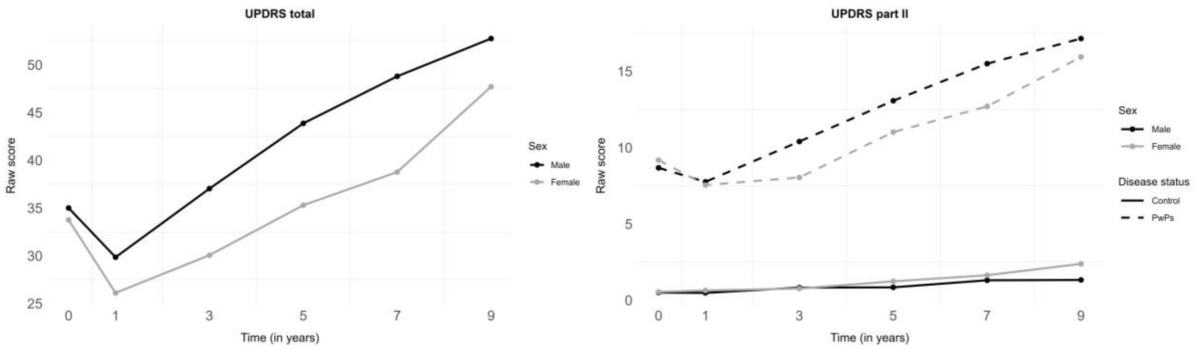

Figure S1. Unified Parkinsons Disease Rating Scale. Graphic illustration of UPDRS part II and total score-trajectory over 9 years in males and females, with and without PD.

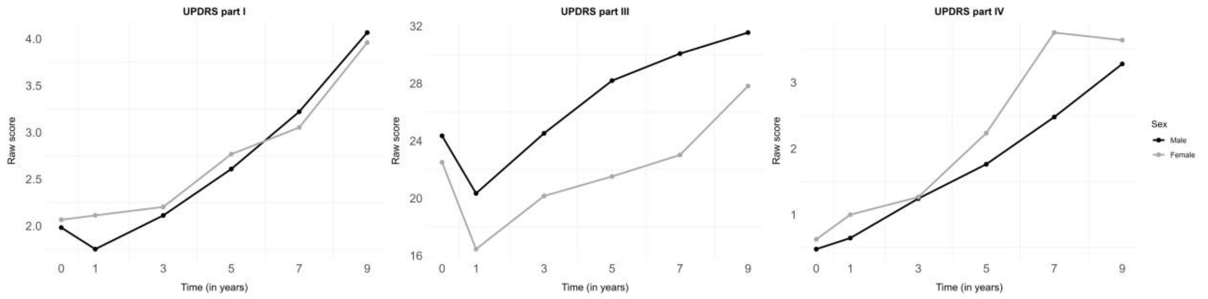

Figure S2. Unified Parkinsons Disease Rating Scale**.** Graphic illustration of UPDRS part I, III and IV score-trajectory over 9 years in male and female PwPs.

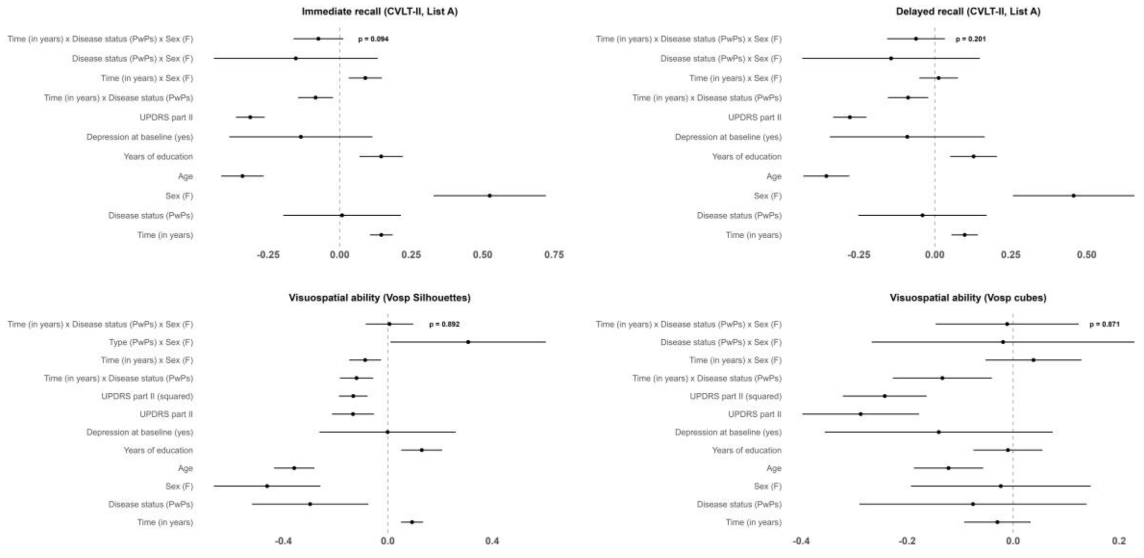

Figure S3. Scaled coefficient plots**.** Scaled output of linear mixed-effect models (CVLT-II and Vosp).

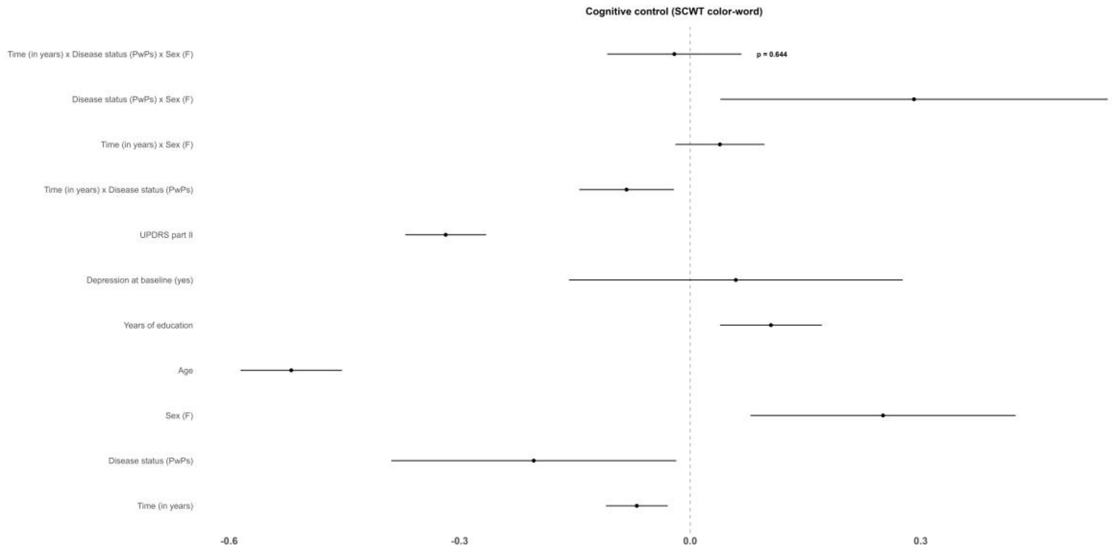

Figure S4. Scaled coefficient plot**.** Scaled output of linear mixed-effect model (SCWT colour-word).

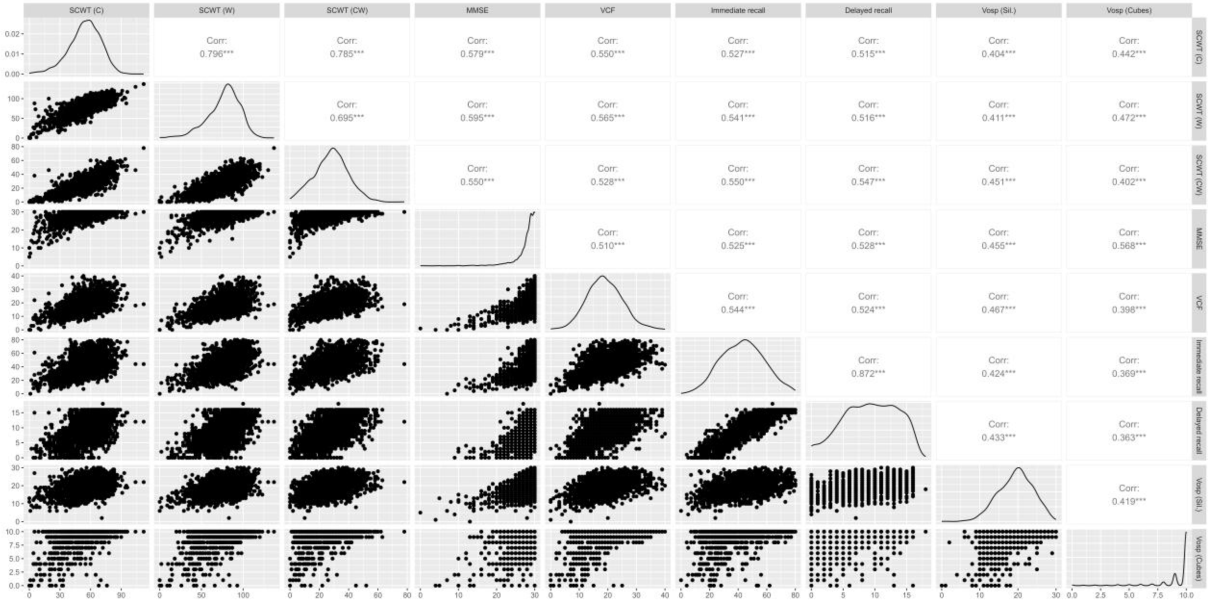

Figure S5. Pairwise correlations. Pairwise correlations of the cognitive test battery.

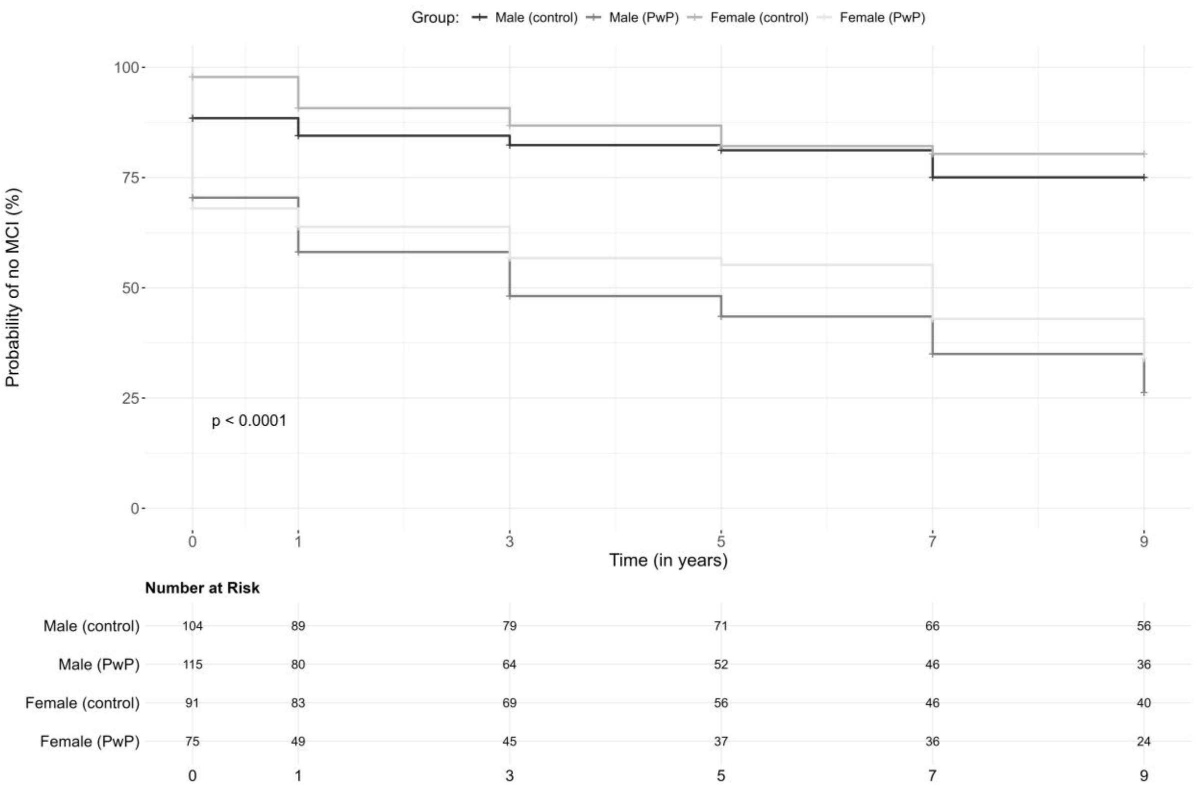

Figure S6. Kaplan-Meier curve. The share of males and females with and without Parkinson’s disease without mild cognitive impairment (MCI).

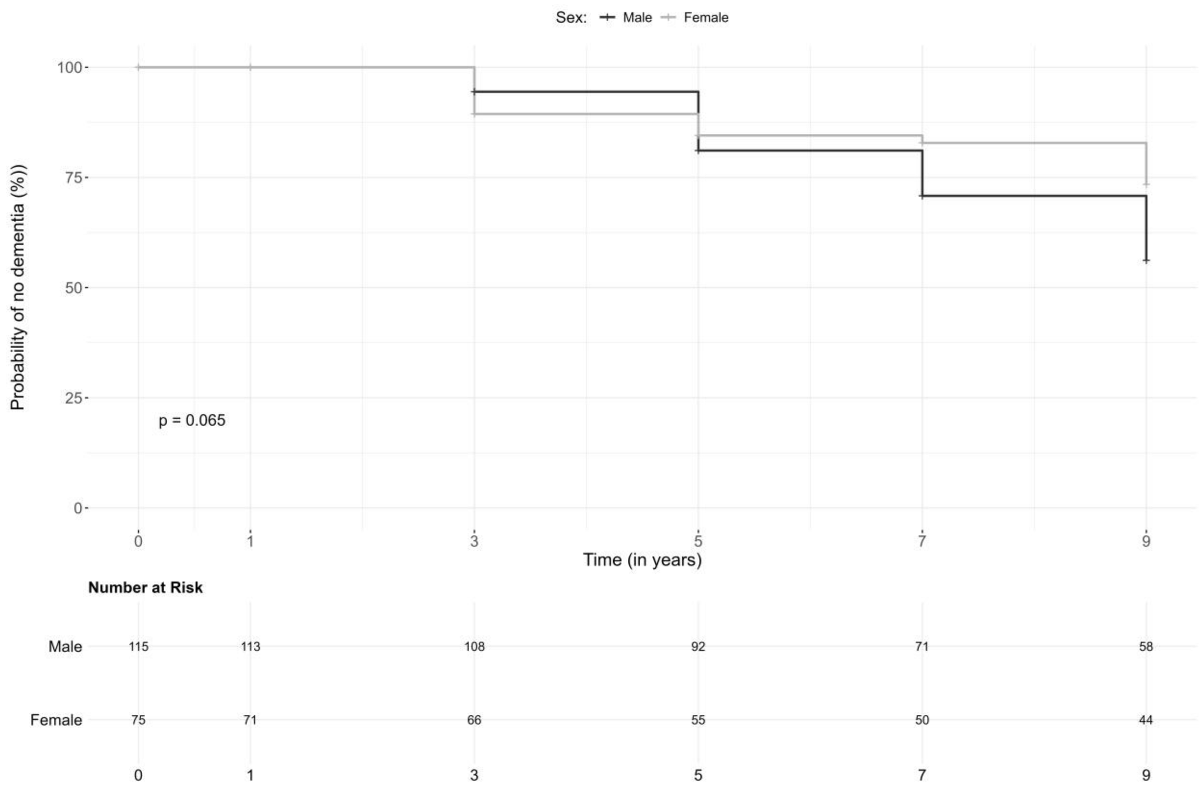

Figure S7. Kaplan-Meier curve. The share of male and female PwPs with no Parkinson's disease associated dementia.
